# Nuclear Factor Erythroid 2-Related Factor 2 (NRF2) as a Biomarker for Radiation Dosimetry and Health Risk Assessment: A Review

**DOI:** 10.3390/antiox14121393

**Published:** 2025-11-22

**Authors:** Kave Moloudi, Traimate Sangsuwan, Satoru Monzen, Yohei Fujishima, Donovan Anderson, Benjamin Frey, Tomisato Miura, Samayeh Azariasl, Hiroshi Yasuda, Siamak Haghdoost

**Affiliations:** 1 Laser Research Centre, Faculty of Health Sciences, Doornfontein Campus, University of Johannesburg, Johannesburg 2028, South Africa; kave.moloudi@unicaen.fr; 2 Laboratoire Aliments, Bioprocédés, Toxicologie Environnements (ABTE, UR4651), University of Caen, Normandy, Cedex 04, F-14050 Caen, France; traimate.sangsuwan@unicaen.fr; 3 Advanced Resource Center for HADrontherapy in Europe (ARCHADE), F-14000 Caen, France; 4 Department of Health Sciences, Hirosaki University Graduate School of Health Sciences, Hirosaki University, Aomori-ken 036-8560, Japan; monzens@hirosaki-u.ac.jp; 5 Department of Risk Analysis and Biodosimetry, Institute of Radiation Emergency Medicine, Hirosaki University, Aomori-ken 036-8560, Japan; yohei.fujishima@hirosaki-u.ac.jp (Y.F.); ande4163@hirosaki-u.ac.jp (D.A.); tomisato@hirosaki-u.ac.jp (T.M.); 6 Translational Radiobiology, Department of Radiation Oncology, Universitätsklinikum Erlangen, Friedrich-Alexander-Universität Erlangen-Nürnberg, 91054 Erlangen, Germany; benjamin.frey@uk-erlangen.de; 7 Department of Radiation Biophysics, Research Institute for Radiation Biology and Medicine, Hiroshima University, Kasumi, Minami-ku 734-8553, Japan; d220190@hiroshima-u.ac.jp (S.A.); hyasuda@hiroshima-u.ac.jp (H.Y.); 8 Department of Molecular Biosciences, The Wenner-Gren Institute, Stockholm University, SE-10691 Stockholm, Sweden

**Keywords:** biodosimetry, NRF2 factor, whole-body exposure, biological modeling

## Abstract

Nuclear factor erythroid 2-related factor 2 (NRF2) is a key transcription factor that controls the antioxidant response to oxidative stress, especially after exposure to ionizing radiation (IR). This review examines NRF2’s emerging role as a complementary biomarker in radiobiological dosimetry for assessing radiation exposure and its potential health effects. When cells encounter IR, the resulting reactive oxygen species (ROS) interfere with the NRF2 repressor KEAP1, leading to NRF2 activation and the expression of cytoprotective genes such as *HO-1*, *NQO1*, and *GCLC*. Evidence suggests that NRF2 levels increase in a dose- and time-dependent manner, primarily at low to moderate radiation doses, highlighting its potential for early detection of radiation exposure. However, at high doses (>8 Gy), NRF2 activation may be suppressed due to apoptosis or irreversible damage, which limits its reliability in those situations. The review also compares NRF2 with other biomarkers used in biodosimetry, discussing its advantages, such as sensitivity and early response, along with its limitations, including variability in activation at high doses and expression influenced by other oxidative factors. The authors introduce a comprehensive radiobiological model that illustrates how low-dose IR exposure affects NRF2 expression patterns, thereby improving the understanding of dose-dependent oxidative stress mechanisms. Additionally, the role of NRF2 in inflammation and general health risk assessment is emphasized, suggesting broader applications beyond biodosimetry. Overall, NRF2 holds significant promise for use in evaluating radiation exposure, developing radioprotection strategies, and informing future radiobiological research frameworks.

## 1. Introduction

Biological dosimetry (biodosimetry) is a crucial method for measuring radiation exposure, especially in emergencies [1]. Although well-established for external radiation, interpreting biodosimetry data for internal exposures still presents difficulties [2,3]. Several techniques, including cytogenetic assays, have proven useful for measuring radiation effects in patients and estimating doses to assess cancer risk [4]. Using multiple biological markers within a complex biodosimetric system can lead to more precise dose estimates, particularly when physical dosimetry data is not available [5]. Biodosimetry methods are applied not only in radiation protection but also in clinical practice and in assessing different medical exposure modalities [6]. Ongoing research seeks to enhance biodosimetry techniques through automation, molecular markers, and multiparametric platforms. Biodosimetry techniques are mainly classified into two categories: biology-based and physics-based methods. Biology-based methods include assays like dicentric chromosome aberration analysis, cytokinesis-block micronucleus (CBMN) testing, fluorescence in situ hybridization (FISH), and assessments of lymphocyte depletion rate (LDR). Conversely, physics-based methods detect radioactivity or radiation-induced free radicals in biological samples, independent of cellular responses. Among these, electron spin resonance (ESR), also known as electron paramagnetic resonance (EPR) spectroscopy, is frequently employed to identify radiation-induced free radicals in tissues such as tooth enamel, bone, and nails, especially in cases of radiological accidents. Due to the stability of these signals over time, ESR/EPR is considered one of the most dependable techniques for retrospective dosimetry [7,8,9].

Recent research emphasizes the improvement of biodosimetry methods for rapid and precise radiation dose assessment during mass casualty events. This includes automating existing techniques, such as the RABiT (Rapid Automated Biodosimetry Tool) system for high-throughput micronuclei analysis [10,11], and using imaging flow cytometry to detect γ-H2AX (phosphorylated H2AX) foci in lymphocytes [12]. Researchers are also exploring molecular markers across genomics, proteomics, metabolomics, and transcriptomics to enhance biodosimetry capabilities [13]. Combining various assays, like γ-H2AX foci, dicentrics, and translocations, into multiparametric approaches is proposed to improve dose estimation accuracy, especially for low doses [14]. Development of field-ready technologies, such as in vivo EPR dosimetry [15], along with establishing laboratory surge capacity networks, aims to strengthen biodefense preparedness [16,17]. These advances aim to address the limitations of current, time-consuming, and labor-intensive biodosimetry methods, thereby facilitating rapid, individualized dose assessments in radiological emergencies [18].

Research has identified several potential biomarkers, including oxidative stress indicators like parkin and NRF2 (Nuclear factor erythroid 2 2-related factor 2), which demonstrate high sensitivity and persistence [19,20]. Other promising markers are γ-H2AX, microRNA, lncRNA, and 8-Oxo-dG [21]. Gene expression and protein markers in peripheral blood have been studied for early detection of acute radiation syndrome, considering their responses to different radiation qualities and time-dependent changes [22]. In irradiated mice, dose-dependent increases in *NRF2* target gene expression have been noted, with ferritin heavy polypeptide 1 (Fth1) showing a strong positive correlation with radiation dose. Moreover, glutathione reductase (GSR) expression is linked to various radiation-induced damages, indicating its potential use as a biodosimeter and damage marker [23]. Transcriptomic approaches show promise for radiation dose reconstruction and injury prediction, although challenges remain in managing individual variability and confounding factors [24]. A key challenge in employing NRF2 target gene expression for biological dosimetry is ensuring its specificity, sensitivity, and measurement standardization across various radiation types, doses, and time points [25]. This review emphasizes recent findings of NRF2 in biodosimetry and health assessment, along with several suggestions for future research.

## 2. Biodosimetry (History, Advantages, and Disadvantages)

Biodosimetry, developed over the past six decades, remains a valuable method for assessing radiation exposure [26]. Back to the late 1960s, following the atomic bombings and early studies on radiation-induced biological effects [27,28,29]. The discovery that ionizing radiation (IR) causes chromosomal damage led to the development of cytogenetic assays, with the dicentric chromosome assay (DCA) becoming the gold standard by the 1960s [30]. DCA is considered the gold standard in biodosimetry because it meets three essential criteria for biomarkers in this field: (1) radiation specificity, (2) stability, and (3) dose dependence. Among these, stability presents a particular challenge for applications in cases of unexpected or delayed exposure assessment.

DCA relies on analyzing metaphase chromosomes, which becomes more difficult at high doses when the G2-to-M transition is suppressed. This limitation was partly overcome by discovering that premature chromosome condensation (PCC) could be induced through cell fusion techniques with viruses or polyethylene glycol [31,32]. Later, PCC was shown to be inducible in dividing cells by using phosphatase inhibitor treatment [33,34]. These technical advances enabled the cytogenetic analysis of peripheral blood lymphocytes from patients exposed to lethal radiation doses, which had previously been challenging [35]. Despite these developments, challenges remain in reliably evaluating low-dose radiation exposures (doses between 0 and 2 Gy) and the development of truly high-throughput analysis methods remains challenging.

In the following decades, advances in molecular biology introduced gene expression markers and biochemical indicators as alternative methods for radiation biodosimetry. More recently, the field has adopted high-throughput technologies, such as the RABiT system, and omics-based approaches, which have improved the speed, automation, and scalability of biodosimetry for potential mass-casualty radiological events [36,37,38]. The cytokinesis-block micronucleus (CBMN) assay has also become a standardized technique for assessing in vivo radiation exposure. Compared to DCA, CBMN has the advantages of being more cost-effective and faster [39,40,41]. However, its sensitivity is limited by variability in background micronucleus frequency, especially at low doses

Additionally, the FISH-based translocation (FISH-Tr) assay has been developed as a complementary cytogenetic method [42]. Unlike dicentrics, stable translocations remain for decades after exposure, making the FISH-Tr assay especially useful for retrospective biodosimetry and long-term dose reconstruction [43,44]. Its main disadvantages include higher cost, technical complexity, and limited availability compared to standard cytogenetic tests; however, its persistent signal offers unique benefits in epidemiological research and the follow-up of chronically exposed populations.

Chromosome aberration scoring remains the most reliable method for quantifying individual exposure and may also aid in personalized radiotherapy planning by identifying individuals who are radiosensitive [45]. However, biodosimetry is essential when physical dosimeters are unavailable or inadequate. While cytogenetic assays, such as DCA, PCC, CBMN, and FISH-Tr, are still commonly used, emerging molecular and high-throughput techniques are revolutionizing biodosimetry, enabling faster, more scalable, and more personalized assessments of radiation dose [46,47]. The pros and cons of biodosimetry are summarized in Table 1. Importantly, both cytogenetic and molecular biomarkers mainly detect direct DNA or chromosomal damage. Growing evidence suggests that indirect effects of ionizing radiation—especially those mediated by oxidative stress and inflammatory responses—also significantly influence radiation sensitivity and long-term health outcomes. In this context, the transcription factor NRF2 has garnered considerable attention for its key role in antioxidant defense and inflammatory response [48], making it a promising biomarker in radiobiological dosimetry and health effects [49].

## 3. Mechanism of NRF2 Activation in Radiobiology

**Figure 1:**
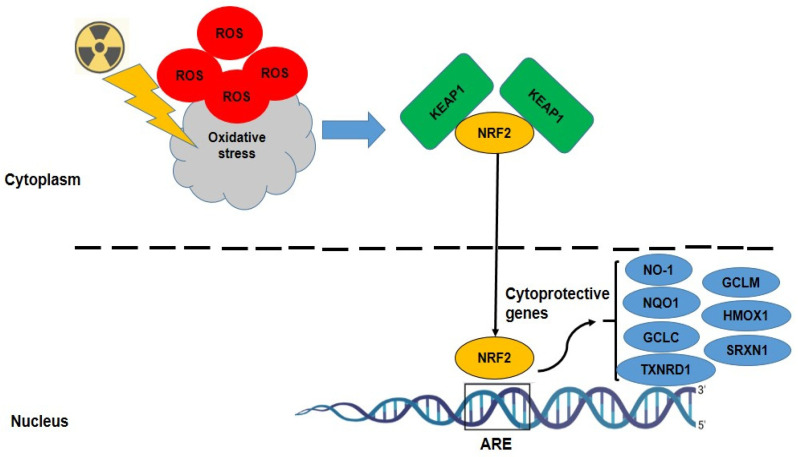
IR triggers NRF2 activation, which subsequently upregulates several cytoprotective genes, including GCLC (Glutamate–Cysteine Ligase Catalytic Subunit), GCLM (Modifier Subunit), HMOX1 (Heme Oxygenase 1), NQO1 (NAD(P)H Quinone Dehydrogenase 1), SRXN1 (Sulfiredoxin 1), and TXNRD1 (Thioredoxin Reductase 1).

NRF2 is a transcription factor that becomes activated in response to oxidative stress and regulates the expression of genes involved in the antioxidant defense system, as shown in Figure 1. Several studies have demonstrated that NRF2 and its target genes can be dose-dependently activated by IR [23,50,51]. This activation occurs after a delay and may contribute to radiation resistance [50]. NRF2 and its downstream targets, such as parkin and heme oxygenase-1 (*NO-1*), have been proposed as potential biomarkers for radiation dosimetry and damage assessment [19]. A panel of robust NRF2 target genes, including *NO-1*, *GCLC*, *GCLM*, *HMOX1*, *NQO1*, *SRXN1*, and *TXNRD1*, has been identified across multiple cell types and species [52,53,54]. Table 2 summarizes the role of cytoprotective genes regulated by NRF2. While NRF2 activation appears to be essential for maintaining radiation resistance, its role as a biomarker in radiobiological dosimetry requires further investigation [23]. Several clinical trials have reported that tumors with constitutive NRF2 pathway activation—most commonly via KEAP1 loss or NFE2L2 mutation—are repeatedly associated with poorer local control and radio-resistance, especially in non-small-cell lung cancer. This signal has prompted ongoing translational and clinical efforts to validate KEAP1/NFE2L2 as predictive biomarkers for radiotherapy outcomes and to develop noninvasive readouts and combination strategies to overcome NRF2-driven resistance, such as PET imaging to report tumor redox/xCT activity and various biomarker/therapeutic trials enrolling NRF2-altered tumors [55,56,57].

## 4. In Vitro, In Vivo, and Clinical Studies

Several studies suggest that NRF2 is a key marker for indicating oxidative stress and IR dose in biological tissue (Table 3). For example, low-dose gamma radiation can influence tissue responses to subsequent high-dose exposure. Pre-exposing tissues to 40 mGy gamma radiation before a 4 Gy dose reduced oxidative stress, DNA damage, and apoptosis in rat liver and testis tissues [58,59]. A recent study by Bradfield et al. demonstrated that whole-body irradiation of mice with 60Co at 7.9 Gy (LD90/30) and 6.85 Gy (LD50/30) increased ferritin, HO-1, and inflammatory cytokine production in the liver, with peak levels observed around day 21 [60]. Additionally, Fréchard and colleagues reported that inhaled tungsten combined with low-dose radiation (50 mGy) caused severe, persistent, and region-specific neurotoxic effects in the brain compared to either stressor alone. The observed changes in oxidative stress pathways and microglial activity suggest a complex mechanism involving NRF2-mediated redox imbalance, neuroinflammation, and microglial redistribution from 24 h to 28 days. Cameron et al. reviewed that NRF2 and related cytoprotective proteins increase after IR injury in various organ systems, including the gastrointestinal (GI) tract, lungs, skin, and bone marrow [61]. Gamma radiation (~0.1 Gy) was found to activate NRF2 and promote its translocation to the nucleus in mouse macrophages via the ERK1/2 pathway after 24 h [62]. An additional study indicated that oxidative stress markers, such as parkin and NRF2, are more sensitive and persistent than nuclear DNA damage, making them potential biomarkers for radiation dosimetry. Shimura et al. demonstrated that a single 5 Gy whole-body X-ray dose significantly increases DNA damage (γ-H2AX), parkin, and NRF2 in mouse blood cells, triggering sustained oxidative stress responses in peripheral lymphocytes starting at 24 h and peaking between 48 and 72 h post-irradiation [19]. Liu and colleagues assessed the temporal changes in NRF2 after an acute 6 Gy dose using targeted mass spectrometry at 1, 2, 3, and 4 days [25]. Their findings showed a strain- and sex-dependent pattern in NRF2-mediated antioxidant responses. For instance, C57Bl/6 males showed increased levels of CAT, SOD1, and HO-1 proteins peaking between days 2 and 3, while GSTM1 consistently decreased across all groups post-irradiation. These time-dependent changes suggest that NRF2-related protein expression varies in a delayed but dose-dependent manner, peaking between 24 and 72 h after exposure, and could serve as early biomarkers for acute radiation injury. Another study indicated that a single low dose of 0.02 Gy total-body irradiation in adult mouse long-term hematopoietic stem cells (LT-HSCs), can trigger autophagy and activate the KEAP1 (Kelch-like ECH-associated protein 1) –NRF2 antioxidant pathway by day 6 post-irradiation. This implies that even very low doses of radiation can induce chronic oxidative stress through transient NRF2 activation, which may impair hematopoietic stem cell (HSC) function in the long term and have important implications for low-dose radiation exposure and hematopoietic health [63].

## 5. Comparison of the NRF2 Marker with Other Biological Markers and Methods for Biological Dosimetry

Various biological markers and methods have been compared in Table 4. NRF2 has recently gained attention as a potential biomarker in radiobiological biodosimetry. However, NRF2 helps assess oxidative stress-related effects of radiation, especially at low doses or in cases of chronic exposure scenarios [50]. Unlike direct DNA damage indicators, NRF2 does not detect DNA breaks; instead, it indicates the cellular redox imbalance caused by radiation [68]. Its dose-dependent gene expression and early activation make it suitable for identifying initial biological responses. However, it is less specific to radiation than some traditional markers, as it can also be upregulated by chemical or metabolic stress. Therefore, while NRF2 shows promise for early detection and mechanistic studies, it is most effective when combined with other biodosimetric tools [1,50].

Traditional radiobiological markers provide more direct evidence of radiation-induced cellular damage. For example, γ-H2AX is a well-known marker of DNA double-strand breaks, showing high sensitivity within minutes to a few hours after exposure but with a limited detection window [14,69]. The CBMN, a cytogenetic method, detects chromosomal damage and is commonly used in biodosimetry because of its strong dose–response relationship; however, it requires a longer processing time (3–4 days). Methods like electron paramagnetic resonance (EPR) measure stable radiation-induced radicals in teeth or nails and are helpful for retrospective dosimetry [70]. ESR/EPR and NRF2 activation are complementary tools in radiation biodosimetry. ESR/EPR is ideal for the direct, early detection of free radicals produced by ionizing radiation, offering chemical specificity and high sensitivity. Conversely, NRF2 activation acts as a delayed, indirect marker reflecting the biological response to oxidative stress, including the induction of antioxidant defenses [50]. While ESR/EPR is more suited for physical dosimetry, NRF2-based assays give valuable insights into cellular recovery and long-term radiation effects [71]. Nonetheless, integrating both approaches can enhance the accuracy of dose estimation and deepen our understanding of the mechanisms involved in biodosimetry research.

Compared to γ-H2AX and CBMN, NRF2-based detection is less precise and less robust. Still, it offers valuable insights into radiation-induced oxidative stress, making it a useful complementary marker in integrated biodosimetry platforms.

**Table 4:** Comparison of the NRF2 factor and its biomarkers with other methods of biodosimetry.

Method	Principal and Biomarker	Range of Doses	Best Time After Exposure (h)	References
Dicentric chromosome assay (DCA)	Unstable chromosomal aberrations	0.1–5 Gy	48–72 h	[72,73,74]
Premature chromosome condensation (PCC) assay	Unstable chromosomal aberrations	0.2 to 20 Gy	For fusion PCC is 2–6 h/for chemical PCC is 40–72 h	[75]
Fluorescence in situ hybridization (FISH) translocation assay	Stable chromosomal aberrations	0.25–4 Gy	48–72 h (to obtain metaphases), but stable translocations can be detected months to years later	[76]
Cytokinesis-block micronucleus (CBMN) assay	Micronuclei in binucleated cells	0.2–4 Gy	~48–72 h	[77,78]
γ-H2AX foci	DNA double-strand break marker	<0.1–3 Gy	0.5–6 h (ideal 0.5–1 h) (functional up to ~24 h)	[14,69]
ESR/EPR	Detects unpaired electrons in radicals or paramagnetic species	0.1–9 Gy	Depends on material: radicals in soft tissues/fingernails—hours to days; tooth enamel or bone—any time (months–years)	[70,71]
NRF2 activation	Antioxidant gene activation (*HO-1, NQO1*)	0.02–8 Gy	~2–24 h (common peak 4–12 h)	[1,50]

## 6. A Prediction Radiobiological Model of NRF2 Expression

**Figure 2:**
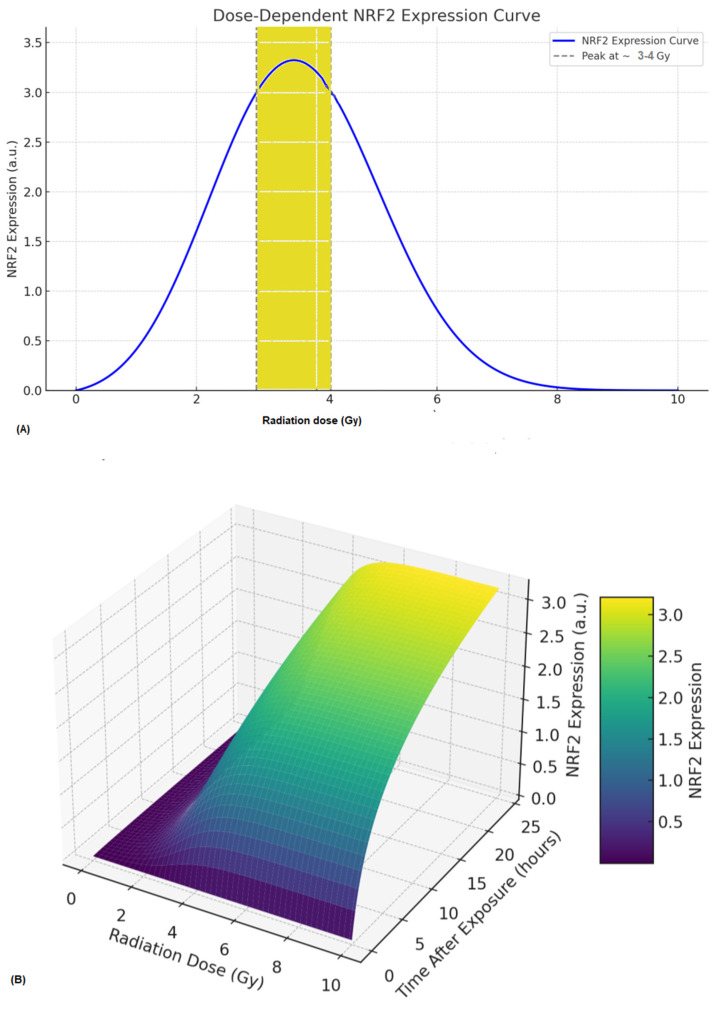
The programmatically generated NRF2 expression curve in response to increasing radiation dose. (**A**), the curve rises sharply at low doses (~0−2 Gy) due to stress-induced NRF2 activation and peaks around 3−4 Gy (the yellow area which optimal ROS induction for NRF2 stabilization)**,** then declines at higher doses (>5 Gy), reflecting suppression due to overwhelming oxidative damage or apoptosis. (**B**), a 3D curve illustrates how NRF2 expression varies based on both time and the dose of IR. (Created by ImageJ, version: 1.54p).

Based on the literature, we have proposed a predictive radiobiological model of NRF2 expression that aims to describe how NRF2 responds to IR based on dose, time, and biological processes. The conceptual and partly mathematical outline of this model is suitable for research or simulation purposes. It estimates changes in NRF2 levels in cells following IR exposure by considering three key factors: (1) dose–response relationship, (2) time after exposure, and (3) cellular oxidative stress response and regulation via KEAP1. According to the model, NRF2 expression increases in a dose-dependent manner (from 0.5 to 4 Gy), then decreases by 8 Gy. However, NRF2 expression exhibits a bell-shaped pattern over time after radiation exposure (Figure 2). At 0.5 Gy, activation is low, peaking between 4 and 6 h and lasting about 12 h. At 2 Gy, expression reaches a moderate level, with the peak delayed to 6–8 h and activity extending up to 18 h. A 4 Gy dose causes a strong NRF2 response, peaking at 8–10 h and remaining active for approximately 24 h. Additionally, at 8 Gy, NRF2 expression diminishes or is suppressed, indicating a potential breakdown of the oxidative stress response system at high doses, where excessive cellular damage may inhibit regulatory pathways. Based on the literature, the expression of NRF2 after IR exposure can be modeled by the equations and biological frameworks (a & b) outlined below.

(a) dN(t)/dt = *β* × ROS(t) × (1 − N(t)/N_max_) − ˠ N(t)
  where N(t) = The quantity or level of NRF2 at time (t), *β* = rate of NRF2 activation by ROS, γ = degradation rate of NRF2 and N_max_ = maximum NRF2 expression capacity.
(b) ROS = αD × e^λ1t^
  where D = Absorbed radiation dose (Gy), α = scaling factor, λ_1_ = ROS clearance rate. However, ROS generation increases with dose and decays over time [79,80]. Furthermore, the observed non-linear activation of NRF2 within the 0.5–4 Gy range likely reflects the complex interplay between direct DNA damage signaling and secondary oxidative stress responses. At lower doses (<1 Gy), transient ROS production induces modest NRF2 nuclear translocation, whereas higher doses (≥3–4 Gy) can suppress NRF2 activity through the oxidative degradation of KEAP1 or overwhelming cellular stress, resulting in a biphasic pattern. Several studies [19,23,50] support this non-linear dose dependence in human PBMCs and fibroblasts. Moreover, the NRF2 activation window (6–24 h) varies with cell type, redox status, and radiation quality, and high-LET radiation or metabolically active cells often exhibit earlier and more sustained activation.

## 7. Use of NRF2 Signaling as a Marker for Radiation-Induced Chronic Oxidative Stress and Chronic Inflammation

### 7.1. NRF2 as a Marker for Radiation-Induced Oxidative Stress

NRF2 indicates radiation-induced oxidative stress in preclinical models. In vitro and in vivo studies show that NRF2 activation can happen immediately [19,81] or after a delay of several days [50,68], with timing differing by cell type and radiation dose (Table 5). NRF2 activity seems to increase with irradiation, evidenced by dose-dependent upregulation of target genes like *Fth1* and heightened ARE-driven transcription, as reported by Miura et al. [23]. Several studies also link NRF2 activation to radioprotection. For example, Singh et al. observed a reduction in cell survival from 27% to 2–3% at 6 Gy following NRF2 suppression. Kim et al. further showed a dose-modifying factor of 1.61 after NRF2 activation with bardoxolone methyl. These results support NRF2 as a sensitive marker for radiation-induced oxidative stress in preclinical settings [82]. While these studies suggest NRF2 could serve as a biomarker for radiation-induced oxidative stress, findings are based on preclinical models with different methodologies and endpoints.

### 7.2. NRF2 as a Marker for Radiation-Induced Inflammatory Response

IR triggers a linked oxidative–inflammatory response where early ROS activate stress kinases and transcription factors [87], especially NF-κB, leading to cytokine release (TNF-α, IL-1β, IL-6). NRF2 serves as a key redox regulator that both detects this oxidative environment and reduces inflammation by inducing cytoprotective enzymes (HMOX1/HO-1, NQO1, GCLC/GCLM, TXNRD1) and by counteracting NF-κB signaling through redox buffering and HO-1–derived mediators. In vivo, whole-body IR increases NRF2 target levels (HO-1, ferritin) along with inflammatory cytokines, peaking at about 24–72 h, supporting NRF2-axis markers as early signs of radiation-induced inflammation [51,88].

**Figure 3:**
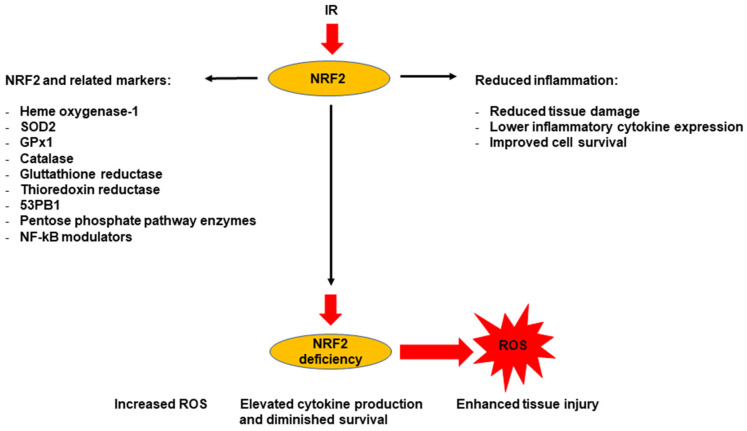
NRF2 functions as a protective marker against radiation-induced inflammation.

NRF2 and its target genes—including heme oxygenase-1, *SOD2*, *GPX1* (Glutathione peroxidase 1), catalase, glutathione reductase, thioredoxin reductase, *53BP1*, enzymes of the pentose phosphate pathway, and NF-κB modulators—serve as markers indicating decreased inflammatory responses caused by radiation [89]; see Figure 3. The activation of NRF2 following IR is linked to less tissue damage, reduced inflammatory cytokine levels, and enhanced cell survival [90].

Mechanistically, low-to-moderate γ-rays quickly promote NRF2 nuclear translocation in macrophages through ERK1/2 within 24 h, creating a cell-intrinsic link between dose-dependent oxidative stress and inflammatory programming [62]. Increasing doses result in higher levels of NRF2 targets, including Fth1, HMOX1, Nqo1, and Gsr, in irradiated mouse blood, which correlate with tissue injury markers—making these transcripts and proteins useful as inflammation-related biodosimeters [23]. Beyond immediate signaling, organ studies demonstrate that the loss of NRF2 worsens radiation-induced inflammatory injury, such as in the lung, while NRF2 activity provides protection [91,92]. Recent crosstalk reviews summarize these findings, describing direct and indirect NRF2–NF-κB interactions that affect the intensity and duration of post-irradiation inflammation [93].

A compact “NRF2-inflammation panel” in peripheral blood measured at 6, 12, and 24 h post-exposure, utilizing qPCR and protein analysis for HMOX1, NQO1, FTH1, GCLC/GCLM, TXNRD1 (±SLC7A11), co-assayed with TNF-α, IL-1β, IL-6, and CCL2, captures the temporal relationship between oxidative stress and inflammatory signaling. Although not radiation-specific, the dose–response behavior, defined detection window, and mechanistic basis—such as myeloid NRF2 translocation within 24 h and target-gene peaks between 24 and 72 h—support the use of NRF2-axis markers as complementary inflammatory readouts alongside γ-H2AX/CBMN for triage and injury assessment [62].

In several experimental models, the genetic overexpression or pharmacological induction of NRF2 leads to delayed antioxidant responses, which decrease ROS and inflammatory signaling. In contrast, NRF2 deficiency consistently correlates with increased ROS, higher cytokine production, greater tissue damage, and lower survival (Table 6). Genes regulated by NRF2 emerge as key markers of this radioprotective response [94]. Notably, heme oxygenase-1 appears in three studies, while other targets such as 53BP1, SOD2, GPX1, catalase, glutathione reductase, thioredoxin reductase, enzymes of the pentose phosphate pathway, and modulators of NF-κB vary in significance across models [95]. However, as reported in some studies (Table 6), in the lung, crypts, hematopoietic tissues, and immune cells, NRF2 and its targets are linked to improved repair and regeneration after irradiation, though certain contexts reveal variable responses.

However, it is important to recognize that low-dose radiation combined with immune checkpoint blockade can induce ferroptosis through the NRF2/HO-1/GPX4 signaling pathway, leading to an inflammatory antitumor response [96]. Additionally, evidence shows that excessive activation of NRF2 promotes cancer cell growth and proliferation, while also increasing resistance to chemotherapy and radiation [97]. These factors should be carefully considered in biodosimetric applications to avoid misinterpreting the results. Ultimately, these are further indications that NRF2 is not limited to controlling and resolving oxidative stress in inflammation [98].

**Table 6:** NRF2 target genes and proteins as inflammatory markers.

NRF2target	Tissue/Cell Line	Radiation Response	Inflammatory Role/Inflammation Time Response	References
*HO-1*	Fibroblasts, breast cancer cells	Upregulated after radiation; absent in Nrf2-deficient cells	Antioxidant, cytoprotective/early response (hours to 1 day)	[50,59]
*HO-1*, *p53-binding protein 1* (*53BP1*)	Colonic epithelium, crypts	Increased DNA repair, reduced apoptosis, improved survival	Anti-inflammatory, DNA repair/early (hours)	[85]
Glutathione reductase (*GR*), thioredoxin reductase 1 (*TRXR1*), pentose phosphate pathway (*PPP*) enzymes, nuclear factor kappa B (*NF-κB*)	Mouse embryonic fibroblasts, immune cells	Reduced transformation, lower NF-κB activation in wild-type	Antioxidant/early to intermediate (hours to days); NF-κB is often activated within hours	[68]
*GPX1*, *SOD2*, *CAT*, *HO-1*	Lung	Reduced oxidative damage, lower pro-inflammatory cytokines, higher interleukin-10 (IL-10)	Antioxidant/early to intermediate (hours to a few days); antioxidant enzymes respond early to ROS	[91]
CDDO targets, delta Np63 (ΔNp63)	Crypts, lung	Attenuates crypt injury, modulates stem cell response	Modulates ROS, transforming growth factor beta (TGF-β)/Smad, collagen degradation/Intermediate (days)	[61]
SOD1, 53BP1, plasminogen activator inhibitor-1 (PAI-1)	Lung, bone, glioblastoma	Promotes DNA repair, detoxifies ROS, suppresses fibrosis	Modulates cytokines, suppresses TGF-β1	[94,99]
NRF2 promotes radiation resistance by cooperating with TOPBP1 to regulate DNA repair	Human lung cancer cell lines (radioresistant derivatives, e.g., A549/A549R) and mouse xenografts	Evaluation of NRF2 protein, chromatin fractionation, functional assays (clonogenic survival), and γ-H2AX	Altered inflammatory gene expression in the tumor microenvironment in models, linking NRF2 to both radioresistance and radiation-associated inflammatory signaling.	[100]

## 8. A Summary on the Role of NRF2 as a Biomarker for Health Risk Assessment

NRF2 has become a promising biomarker for health risk assessment, with PBMCs serving as an accessible source for monitoring NRF2 activation in clinical settings [48,101]. Clinical trials testing NRF2-targeting compounds, such as dimethyl fumarate, bardoxolone methyl, oltipraz, and sulforaphane, have shown that these agents influence the NRF2 pathway in humans. However, no single biomarker is ideal for defining pharmacodynamic actions [102]. Computational approaches have identified a 143-gene biomarker with 93% balanced accuracy for predicting NRF2 activity, including well-known target genes like *NQO1*, *GCLC*, and *TXNRD1* [103]. A comprehensive review of the literature has identified *GCLC*, *GCLM*, *HMOX1*, *NQO1*, *SRXN1*, and *TXNRD1* as a reliable panel of NRF2 biomarkers that are directly regulated across various cell types, offering a standardized method for evaluating NRF2 signaling in translational research [54]. Nevertheless, NRF2 is a vital biomarker for health risk assessment because it reflects the cell’s ability to combat oxidative stress and environmental insults by activating antioxidant and cytoprotective pathways.

**Figure 4:**
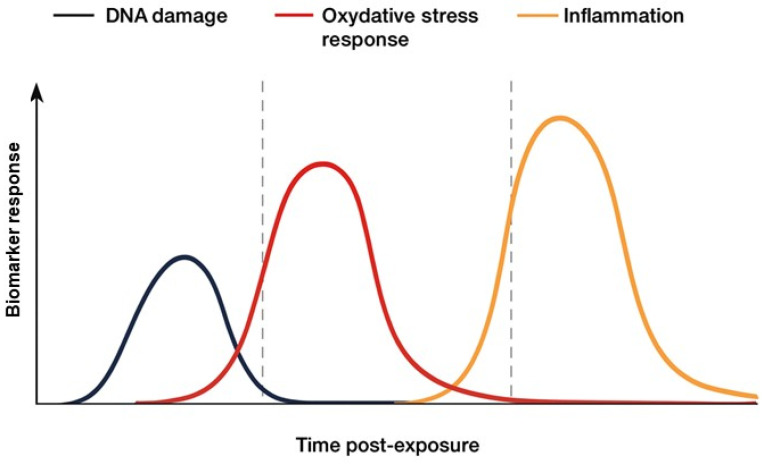
The timing of biomarker responses post-exposure depends on the radiation dose. DNA damage markers may appear as early as 30 min after exposure, whereas inflammatory biomarkers can take several hours to several days to become detectable. Concordant responses across all biomarker classes may indicate acute or high-dose exposure, while discordant patterns (NRF2 activation without detectable DNA damage) may reflect sub-toxic oxidative stress or adaptive responses.

It is important to note that NRF2 activation is not exclusive to radiation exposure; it can also be triggered by chemicals, infections, or metabolic stress [98,104]. Therefore, various physiological and lifestyle factors can influence baseline NFE2L2 (NRF2) activity, potentially affecting the interpretation of NRF2-dependent biomarkers. For instance, the circadian rhythm controls the expression of antioxidant enzymes, with NRF2 signaling exhibiting diurnal variation [105,106]. Fasting and nutritional intake also influence redox balance and NRF2 activation, primarily through metabolic stress signaling [107,108]. Similarly, dietary supplements, phytochemicals, and certain medications—including antioxidants, polyphenols, and drugs that modulate redox signaling—can either activate or inhibit NRF2 [109,110]. Recognizing these variables is crucial when comparing NRF2-related outcomes across studies or individuals, as they may act as confounders and should be considered during sample collection, experimental design, or data interpretation. Consequently, it may be beneficial to interpret NRF2-based readouts in conjunction with other biomarkers. For example, established DNA damage indicators such as γ-H2AX or CBMN (with or without FISH) primarily reflect direct radiation-induced DNA double-strand breaks and chromosomal damage. Moreover, while γ-H2AX indicates immediate DNA double-strand breaks, NRF2-based measures provide insights into longer-term cellular oxidative stress and adaptive responses [111,112]. In contrast, NRF2-related redox markers, such as HMOX1, NQO1, GCLC/GCLM, TXNRD1, and FTH1, indicate oxidative stress and downstream antioxidant responses, which may result from radiation-induced ROS or other cellular stressors [98,113]. Integrating DNA damage and redox–response biomarkers provides a more comprehensive understanding of radiation effects, distinguishing primary genotoxic damage from secondary oxidative stress-driven responses. In addition to DNA damage and NRF2-dependent redox markers, inflammatory cytokines such as IL-6, TNF-α, and IL-1β provide further insight into systemic stress and immune activation. Collectively, these three groups of biomarkers—DNA damage (γ-H2AX, CBMN/FISH), oxidative stress response (HMOX1, NQO1, GCLC/GCLM, TXNRD1), and inflammatory cytokines—contribute to a comprehensive assessment of radiation exposure and cellular responses. As Figure 4 shows, when responses are concordant (all markers increased), this may indicate high or acute radiation exposure, causing both direct DNA damage and secondary oxidative or inflammatory stress. Conversely, discordant responses—such as NRF2 activation with no detectable DNA damage—may suggest sub-lethal oxidative stress, adaptive antioxidant responses, or exposure to other stressors. These interactions can clarify how each biomarker group complements the others and their timing, illustrating how NRF2 fits into an integrated biodosimetry panel and enhancing its translational relevance for clinical and research purposes [114,115].

Another key point is that although NRF2 mRNA expression generally remains stable without significant fluctuations, NRF2 protein levels and activity are mainly regulated after translation through phosphorylation and KEAP1-mediated ubiquitination and degradation. Multiple studies provide strong evidence in support of this regulatory mechanism. Specifically, kinases such as PKC, casein kinase 2, and AMP-activated kinase enhance NRF2 activity through phosphorylation, whereas GSK-3βsuppresses it [116]. However, Zheng Sun et al. caution that phosphorylation has a “limited contribution” in modulating NRF2 activity [117]. The use of phosphorylated NRF2 (pNRF2) as a biodosimetry marker remains speculative theoretical, but research strongly advocates for a multi-omics approach that combines transcriptional and post-translational regulation mechanisms [118]. pNRF2 has thus become a functional indicator of pathway activation, and recent studies suggest pNRF2 could be a sensitive biomarker of cellular stress and radiation exposure [119]. These findings underscore the importance of integrating transcriptomic data with proteomic and phospho-proteomic analyses to gain a deeper understanding of NRF2 signaling dynamics and its potential applications in biodosimetry.

## 9. Conclusions and Prospective View

NRF2 acts as a key transcriptional regulator of the cellular antioxidant defense and significantly influences inflammatory responses. Under oxidative stress, it translocates to the nucleus and activates genes such as *HO-1*, *NQO1*, and *GCL* to help restore the redox balance. Additionally, NRF2 suppresses pro-inflammatory pathways such as NF-κB and reduces cytokines like TNF-α, IL-1β, and IL-6. As a biomarker, NRF2 is sensitive to low doses, reflects biological function rather than just dose, and can be measured in blood, tissues, and cultured cells, making it useful for biodosimetry, radiation therapy planning, and exposure assessments. However, limitations include suppression or overwhelm at doses exceeding 4 Gy, a transient activation that peaks within 24 h, and non-specific activation by various stressors, which reduces its diagnostic accuracy for radiation. NRF2 shows a non-linear dose–response: activated at 0.5–4 Gy but suppressed at higher doses due to excessive ROS or damage. Its activation also depends on timing, peaking about 6–24 h post-exposure, so sample collection timing is critical. Tissue differences influence NRF2 responses, complicating interpretation unless sampling is consistent. Since NRF2 does not directly indicate DNA damage, it should be combined with other biomarkers such as γ-H2AX, micronuclei, or p53 for comprehensive biodosimetry. Future strategies may involve integrating multi-omics approaches that involve NRF2 pathways to better capture the effects of radiation. Standardized sampling times, tissue-specific thresholds, and NRF2 pathway modulators could improve diagnostics and therapeutic radioprotection. Advances in high-throughput assays and biosensors could enable NRF2-based panels for early triage, dose estimation, and recovery monitoring after accidental or clinical radiation exposure. Given its sensitivity to blood NRF2 expression following IR, the authors recommend including it in whole-body radiation exposure assessments during emergencies.

## Figures and Tables

**Table 1:** Advantages and disadvantages of biological and physical biodosimetry.

Advantages	Disadvantages
Reflects biological impact (Measures actual damage or cellular response, not just exposure)	Time-consuming (Traditional assays (like DCA) may take days to a week)
Useful when physical dosimeters are absent (Critical for accidents, triage, and emergencies)	Requires lab infrastructure (Needs specialized equipment and trained personnel)
Retrospective assessment (Some methods, e.g., ESR on teeth, allow dose estimation long after exposure)	Variability (Results may vary based on age, sex, health, and genetic background)
Applicable across radiation types (Works for gamma, X-rays, neutrons, and mixed fields)	Limited sensitivity window and unscheduled exposure (Some biomarkers are transient and require fast sampling post-exposure)
Multiple biomarkers available (Allows customization (cytogenetic, molecular, biochemical) depending on timeframe and context)	Low throughput (Many conventional methods are not scalable for mass casualty events)
Useful for internal and organ dosimetry	Not always dose-specific (Some markers may be non-specific to radiation) (e.g., NRF2 also responds to oxidative stress from non-radiation sources)

**Table 2:** Cytoprotective genes regulated by NRF2.

Genes	Function	Antioxidant Role
*GCLC*	GSH synthesis (catalytic)	Maintains redox balance
*GCLM*	GSH synthesis (modifier)	Enhances GCLC activity
*HMOX1*	Heme degradation	Anti-inflammatory, cytoprotective
*NQO1*	Detoxifies quinones	Prevents ROS generation
*SRXN1*	Restores peroxiredoxins	Supports ROS clearance
*TXNRD1*	Reduces thioredoxin	Supports DNA synthesis, detox

**Table 3:** NRF2 and its markers in vitro, in vivo, and clinical studies.

Radiation Source and Dose	Type of Study	Tissue or Organ	Findings	References
Gamma ray (40 mGy–4 Gy)	In vivo	Liver and testis	Exposure to 40 mGy before 4 Gy induced a significant increase in the levels of NRF2, NRF2 mRNA	[58]
^60^Co (7.9 Gy and 6.85 Gy)	In vivo	Liver	Increased ferritin, HO-1, and inflammatory cytokine	[60]
Tungsten aerosol (80 mg/m^3^) plus low-dose radiation of gamma ray (50 mGy)	In vivo	Brain	NRF2 and pro-inflammatory cytokines (IL-1β and TNF-α)	[64]
Gamma ray (0.1–0.3 Gy)	In vivo	Blood (Mouse macrophage RAW264.7 cells)	NRF2, HMOX1, Ferritin heavy chain (Fth1), Nqo1, GCLC/M, Gsr, and Txnrd1	[62]
X-ray (0.1–5 Gy)	In vivo	Peripheral lymphocytes	Parkin, NRF2, and DNA damage	[19]
Gamma ray (6 Gy)	In vivo	Bone marrow	Upregulation in antioxidant enzymes: NRF2, CAT (catalase), SOD1, and HO-1	[25]
Gamma ray (0 to 2 Gy)	In vivo	Hematopoietic stem cells (HSCs)	NRF2	[63]
X-rays and γ-rays (variable laboratory doses; typically 0.5–5 Gy)	In vitro	Peripheral blood mononuclear cells (PBMCs)	Demonstrated radiation-induced phosphorylation of Serine 360 of SMC1, establishing it as a sensitive molecular marker for radiation exposure.	[65]
X-rays (0.5–6 Gy range)	In vitro (ex vivo human PBMCs)	PBMCs	Specific Genes (*) (*CDKN1A*, *BAX*, *MDM2*, *XPC*, *PCNA*, *FDXR*, *GDF-15*, *DDB2*, *TNFRSF10B*, *PHPT1*, *ASTN2*, *RPS27L*, *BBC3*, *TNFSF4*, *POLH*, *CCNG1*, *PPM1D* and *GADD45A)*	[66]
γ-rays (0.5–8 Gy)	In vitro/translational	PBMCs	Introduced the prematurely condensed chromosome (PCC) assay in PBMCs	[67]

* *CDKN1A* (Cyclin Dependent Kinase Inhibitor 1A), *BAX* (BCL2 Associated X), *MDM2* (E3 Ubiquitin Protein Ligase), *XPC* (XPC Complex Subunit), *PCNA* (Proliferating Cell Nuclear Antigen), *FDXR* (Ferredoxin Reductase), *GDF-15* (Growth Differentiation Factor 15), *DDB2* (Damage-Specific DNA Binding Protein 2), *TNFRSF10B* (Tumor Necrosis Factor Receptor Superfamily Member 10B), *PHPT1* (Phosphohistidine Phosphatase 1), *ASTN2* (Astrotactin 2), *RPS27L* (Ribosomal Protein S27-Like), *BBC3* (BCL2 Binding Component 3), *TNFSF4* (Tumor Necrosis Factor Ligand Superfamily Member 4), *POLH* (DNA Polymerase Eta), *CCNG1* (Cyclin G1), *PPM1D* (Protein Phosphatase, Mg^2+^/Mn^2+^ Dependent 1D), *GADD45A* (Growth Arrest and DNA Damage-Inducible Alpha).

**Table 5:** Various studies reported NRF2 expression after oxidative stress.

Type of Study	Cell Line/Animal	Radiation Dose	NRF2 Assessment Method	Outcomes	References
In vitro, in vivo	Human keratinocytes, SKH1 mice	4 and 30 Gy	NRF2knockdown/activation (bixin), glutathione levels, DNA damage/oxidative stress markers	Radiation-induced dermatitis, DNA damage, oxidative stress, cell viability	[83]
In vitro, in vivo	Primary osteoblasts, C57BL/6J mice	20 Gy	NRF2 knockout, ROS, glutathione (GSH), receptor activator of nuclear factor kappa-Β ligand (RANKL)	Bone loss, osteoblast mineralization, oxidative stress	[84]
In vitro, in vivo	MCF7, C57BL/6 mice	2–8 Gyvia single/fractionated, whole-body	Antioxidant response element (ARE)-dependent transcription, NRF2-deficient vs. wild-type, HO-1	Implicates NRF2 in modulating radiation-induced oxidative stress (with downstream implications for inflammatory responses).	[50]
In vitro	NSCLC, mouse embryonic fibroblasts	0–20 Gy	NRF2 knockdown/overexpression, ROS, antioxidant gene expression	Radioresistance, ROS, cell survival, protein carbonyls	[82]
In vitro	Human rhabdomyosarcoma cell lines	>2 Gy	NRF2 gene expression, silencing, γ-H2AX	Clonogenic survival, ROS, DNA damage, antioxidant response	[81]
In vivo	C57BL/6NCrSlc mice	0.1–5 Gywhole-body	NRF2 immunostaining, parkin, γ-H2AX	Oxidative stress biomarkers, DNA damage, dosimetry	[19]
In vivo	C57BL/6 mice	0.5–3 Gy, whole-body	NRF2 target gene (ferritin heavy chain 1 (*Fth1*), *Gsr* mRNA expression	Dose–response of NRF2 target genes, biological damage	[23]
In vitro, in vivo	Mouse embryonic fibroblasts, C57BL/6 mice	7–8.2 Gy whole-body (mice), 2–8 Gy targeted (cells)	NRF2 knockout, gene expression, ROS, γ-H2AX, immune markers	Transformation, inflammation, radioresistance, immune response	[68]
In vitro, in vivo	Human colonic epithelial cells, *wild-type 129*/*Sv mice*	7.5–10 Gy whole-body	NRF2 activation (bardoxolone methyl (BARD)), ARE binding, HO-1, p53-binding protein 1 (53BP1), DNA repair foci	DNA damage signaling, cell survival, radioprotection	[85]
In vitro	A549 cell line	8 Gy	NRF2 knockout/inhibition, protein localization, ataxia telangiectasia and Rad3-related/checkpoint kinase 1/cell division cycle 2 (ATR/CHK1/CDC2) pathway	ATR activation, G2 arrest, DNA repair, radiosensitivity	[86]

## Data Availability

No new data were created or analyzed in this study. Data sharing is not applicable to this article.
